# Macronutrient and Major Food Group Intake in a Cohort of Southern Italian Adults

**DOI:** 10.3390/antiox7040058

**Published:** 2018-04-15

**Authors:** Serena Mulè, Mariagiovanna Falla, Alessandra Conti, Dora Castiglione, Isabella Blanco, Armando Platania, Maurizio D’Urso, Marina Marranzano

**Affiliations:** 1 Department of Medical and Surgical Sciences and Advanced Technologies “G.F. Ingrassia”, University of Catania, 95125 Catania, Italy; serenamule86@gmail.com (S.M.); mariagiovannafalla@yahoo.com (M.F.); alessandra_conti@ymail.com (A.C.); doracastiglione29@gmail.com (D.C.); dott.ssa.blanco.isabella@gmail.com (I.B.); armplt@hotmail.it (A.P.); 2 Provincial Health Authority of Catania, 95127 Catania, Italy; ma.durso76@gmail.com

**Keywords:** macronutrients, food intake, body mass index, dietary recommendations, cohort

## Abstract

Background: Dietary intake of macronutrient and foods is considered crucial to decrease the risk of diet-related non-communicable diseases. Methods: The aim of this study was to describe the intake of major food groups and macronutrients in a random sample of 1838 southern Italian adults. Results: No significant differences of macronutrient consumption between sexes were found. By contrast, younger individuals had significantly higher intake of animal protein than older ones. Men reported consuming significantly more total processed meats and less eggs than women; egg consumption significantly increased by age groups. Significantly lower intake of fruit in the younger age group compared to older ones was found. Various patterns of correlation between food groups were described. More than half of individuals reached the suggested recommendations for carbohydrate and fiber intake, and about two-thirds met the recommendations for total protein and cholesterol intake, while only a minority met for total fat intake. Total and plant protein, monounsaturated and omega-6 fatty acids, were significantly inversely related with BMI (body mass index), while trans fatty acids and cholesterol were directly correlated. A direct association with unprocessed meats and an inverse association with processed meats was also found. Conclusions: The overall findings suggest that relatively healthy dietary habits are common in southern Italy.

## 1. Introduction

Over the last decades, great efforts have been done to identify a nutritionally balanced diet that might help reduce the risk of chronic non-communicable diseases. There is convincing evidence that dietary factors, alongside with physical activity and abstinence from unhealthy lifestyle behaviors (such as smoking habits), play a crucial role in prolonging the lifespan and ameliorating human health [1,2]. Adequate nutritional requirements represent, nowadays, a key element of public health effort [3]; thus, assessment and knowledge of current populations’ nutritional status is needed to design national recommendations [4]. Previous guidelines were mainly interested in macronutrient intake, but more recent dietary advice focused on food groups, in order to improve the understanding of the general population and facilitate public health educators and policymakers to better identify crucial priorities in the field [5,6].

Research in nutritional epidemiology produced over the last years investigated the association between macronutrients/major food groups, and the most common chronic non-communicable diseases [7]. As prevalence of metabolic disorders has increased in the last decades, major attention has been appointed to the risk of obesity, considered the potential lead mediating factor for many other conditions [8]. In contrast with the individual role of obesity as determinant of diet-related diseases, there is general agreement that calorie source matters, and that diet quality, intended as a proper ratio between macronutrients and individual food groups, constitutes an independent risk factor for negative outcomes [9]. As carbohydrates are generally the most common source of dietary energy, it is therefore intuitive to ascribe to them the major responsibility for higher risk of obesity. However, numerous studies failed in assessing such a relationship, making evidence on this matter difficult to understand [10]. In fact, whether carbohydrates come in the form of whole or refined grains, has been suggested to be relevant in the explanation for the uncertainty of the findings from the studies exploring the association between total carbohydrate intake and weight status [10]. Similar concerns regard dietary guidelines involving protein intake. In fact, there is adequate evidence (from randomized controlled trials, RCTs) showing that substitution of protein for carbohydrate may favorably affect weight management and improve cardiometabolic biomarkers [11,12]. However, the type of protein may have specific effects, and other studies reported that differences between animal and plant protein occur when exploring long-term association with metabolic disorders [13] and overall mortality risk [14,15]. Final important different effects have been recently associated with various dietary fats. The failure of “low-fat diets” in prolonging the lifespan [16] and the discovery of the beneficial effects of (relatively) “high-fat diets”, such as the Mediterranean dietary pattern [17,18], underlined the need to better distinguish between dietary fats and their effects on health. There is evidence that mono- and polyunsaturated fatty acids (MUFA and PUFA, respectively), including omega-3 PUFA from fish and vegetable, may exert a number of beneficial effects compared to saturated or, even worse, trans-fatty acids [19,20]. However, evidence on unhealthy effects of saturated fatty acids, per se, is still controversial, and further research is needed, overall, to better distinguish between subgroups of macronutrients, as aforementioned.

National and international organizations are dealing with current evidence on the association between diet and health. Experts boards continuously draft and update dietary guidelines and recommendations in order to prevent, on a large population scale, common non-communicable diseases. However, data on actual food consumption in cohort studies is often underrated and scarcely described. The aim of the present study was to describe the intake of major food groups and macronutrients in a sample of southern Italian adults, and to analyze the differences in consumption between sexes and age groups. Additionally, the study aimed to explore the correlation between the variables investigated and the association with weight status of participants.

## 2. Materials and Methods 

### 2.1. Study Population

A sample of 2044 men and women aged 18 or more was collected between 2014 and 2016 in the main districts of the city of Catania, southern Italy, to build the Mediterranean healthy eating, ageing, and lifestyle (MEAL) cohort. A detailed description of the study protocol is published elsewhere [21]. Briefly, the theoretical sample size was set at 1500 individuals to provide a specific relative precision of 5% (type I error, 0.05; type II error, 0.10), taking into account an anticipated 70% participation rate. The sampling technique included stratification by municipality area, age, and sex of inhabitants, and randomization into subgroups, with randomly selected general practitioners being the sampling units, and individuals registered to them comprising the final sample units. Out of 2405 individuals invited, the final sample size was 2044 participants (response rate of 85%). All participants were informed about the aims of the study and provided a written informed consent. All the study procedures were carried out in accordance with the Declaration of Helsinki (1989) of the World Medical Association. The study protocol has been approved by the concerning ethical committee (protocol number: 802/23 December 2014).

### 2.2. Data Collection

Data was collected by a face-to-face computer-assisted personal interview using tablet computers. In order to visualize the response options, participants were provided of a paper copy of the questionnaire, however, final answers were filled in by the interviewer directly on the digital device (tablet computer). The demographic and anthropometric data were collected according to standard procedures [22]. Regarding anthropometric measurements, height was measured to the nearest 0.5 cm without shoes, with the back square against the wall tape, eyes looking straight ahead, with a right-angle triangle resting on the scalp and against the wall. Weight was measured with a lever balance to the nearest 100 g without shoes and with light undergarments. Body mass index (BMI) was finally calculated [23].

### 2.3. Dietary Assessment

A food frequency questionnaire (FFQ) previously validated for the Sicilian population was administered to collect information on food consumption [24,25]. The long version of the FFQ used to retrieve the dietary estimates presented in this study consisted of 110 food items; intake of seasonal foods referred to consumption during the period in which the food was available, and then adjusted by its proportional intake in one year. Following the identification of the food frequency consumption, the estimated intakes were converted into daily intake (g/day) and were used to calculate energy and macronutrient content based on online food composition databases (such as the Research Center for Foods and Nutrition CREA—*Consiglio per la ricerca in agricoltura e l’analisi dell’economia agraria*) [26]. Nutrient intake was finally adjusted for total energy intake (kcal/day) using the residual method [27]. FFQs with unreliable intakes (we arbitrarily considered <1000 or >6000 kcal/day as realistically unreliable energy intake; *n* = 107) as well as missing items for the purposes of this study (*n* = 99) were excluded from the analyses, leaving a total of 1838 individuals included in the analysis.

### 2.4. Dietary Recommendations

To investigate agreement with dietary recommendations, we used the European proposed values for macronutrient intake of the European Food Safety Agency (EFSA) [28] and those proposed by the Italian Society of Human Nutrition “*Livelli di Assunzione di Riferimento di Nutrienti”* (LARN) [29], while for major food groups we used the World Health Organization (WHO) recommendations [30].

### 2.5. Statistical Analysis

Frequencies are presented as absolute numbers and percentages; continuous variables are presented as means and standard errors, medians and ranges. Differences between groups for continuous variables were compared with Student’s *t* test and ANOVA for continuous variables distributed normally, and Mann–Whitney U test and Kruskal–Wallis test for variables not normally distributed. Correlations among major food groups were tested through calculation of Pearson’s or Spearman’s correlation coefficients, depending on the distribution of the variable. Linear association between variables of interest and BMI levels were tested through linear regression analyses. All reported *P* values were based on two-sided tests and compared to a significance level of 5%. SPSS 17 (SPSS Inc., Chicago, IL, USA) software was used for all the statistical calculations.

## 3. Results

Table 1 shows the distribution of total energy, macronutrient and fiber intake in the study cohort, by sex and age groups. No significant differences of mean consumption of macronutrients between sexes were found. All macronutrients were mostly equally distributed, and even though men had slightly higher intake of cholesterol and total protein, the difference was not significant compared to women. In contrast, younger individuals consumed significantly more animal protein than older ones.

The description of consumption of major food group from animal source is shown in Table 2. Regarding differences between sex, men reported consuming significantly more total processed meats and less eggs than women (18.00 g/day vs. 14.54 g/day and 2.04 g/day vs. 2.62 g/day, respectively). Difference in egg consumption was also found between age groups, as intake significantly increased with age. Table 3 describes distribution of intake of plant food groups between sex and age groups. No significant differences were evident between sexes, but a significantly lower intake of fruit in the younger age group compared to older ones was found.

The correlation between intake of all major food groups is shown in Table 4. A correlation between fruit, vegetables, legumes, and seafood was found; however, the latter were also correlated with all other animal products, including cheese, eggs, and processed and unprocessed red meats. Whole and refined grain intake was correlated with yoghurt, while nuts and seeds were correlated with both meat and vegetable product intake. However, most of the significant associations were very weak and arguably negligible.

Table 5 describes the percentage of individuals meeting recommendations from LARN, EFSA, and WHO on macronutrients and food group intake. Generally, more than half of individuals reached the suggested recommendations for carbohydrate and fiber intake, while the proportion of adherent individuals was even higher for total protein and cholesterol intake recommendations. By contrast, only a minority met the recommendations for total fat intake.

Table 6 and Table 7 describe the association between macronutrient and major food group intake and BMI levels in the investigated population, total and by sex. Total protein, and specifically plant protein, monounsaturated fatty acids and omega-6 fatty acids were significantly inversely related with BMI, while trans fatty acids and cholesterol were directly correlated (Table 6). However, no significant results were found for major food groups, with the exception of a direct association with unprocessed meats and an inverse association with processed meats (Table 7). It was noteworthy that the magnitude of the latter associations and of proteins, in general, were very small compared to those of dietary fats.

## 4. Discussion

The present study provided updated information on intake of major food groups and macronutrients and their association with weight status in a sample of southern Italian adults. We found that a large proportion of individuals had adequate intake of protein, fiber, fats, fruit and vegetable, meat, and pulses according to national and international recommendations. These results suggest that the investigated population has generally healthy dietary choices; however, investigating major food group consumption and comparison with other reports is crucial to better understand dietary priorities for future strategies to improve dietary habits and overall health.

Despite the importance of monitoring dietary intakes at population level, previous studies investigating macronutrient and food consumption are scarce. A recent report of Global Burden of Diseases Nutrition and Chronic Diseases Expert Group aimed to describe consumption of major food groups worldwide and at national level [31]. Despite that the report showed standardized intake to the same isocaloric diet (2000 kcal/day), our data are comparable, due to similar average total energy intake in both men and women. In 2010, mean global fruit consumption in adults has been reported to be 81.3 g/day, with the highest intake in Greece, and no clear pattern of variation of consumption worldwide. In this study, we reported a much higher fruit intake (about 400 g/day) only comparable with reports from Jamaica and Malaysia. However, two Italian surveys [32,33] showed an average national consumption of fruit closer to those reported in the present study (about 200–300 g/day); our estimates might be higher, due to the higher availability of fruit and lower prices in the regional territory [34] (taking into account that none of the previous reports included the municipality of Catania for sampling), or represent an overestimation, due to potential limitation of this type of recall studies (i.e., higher number of food items coding for “fruit” compared to other FFQs). Mean vegetable and legume consumption in our study was more in line with worldwide average intake (about 250 g/day versus 208.8 g/day, respectively) and those reported in the other Italian studies [32,33]. Moreover, fruit and vegetable consumption were strongly intercorrelated, reflecting a global trend. Consumption of nuts, seeds, and wholegrain is relatively low worldwide (around 10 g/day and 40 g/day), with the highest consumption in Southeast Asian nations and the lowest in Central European nations. Our reports were similar to worldwide average regarding whole-grain consumption, but much higher concerning nut intake (about 20 g/day); again, this can be the result of increased intake due to local production of certain nut subtypes (i.e., pistachios), which might be easier available and at lower price, or an overestimation due to various questions on nut-subtypes in our FFQs. Regarding animal products, in our cohort we found a higher consumption of seafood (about 60 g/day versus 28 g/day), similar of processed meat (about 16 g/day versus 14 g/day), and slightly lower of unprocessed meat (about 34 g/day versus 42 g/day) compared to worldwide reports. However, the higher seafood intake was evident in Pacific Island nations, the Mediterranean Basin, South Korea, and Japan, consistently with historical cultures and local availability. Also, the other Italian report showed similar intake of processed and unprocessed meat products than those reported in the present study, while consumption of fish was lower [32,33]. According to the Italian National Institute of Statistics (ISTAT), the mean expenditure for major food groups in the Italian islands (including Sicily) does not substantially differ from the national average, with the exception of higher purchase of seafood, thus reflecting a regional preference in consuming such products. Interestingly, we have found that seafood intake was weakly correlated with most of the other food groups investigated, suggesting that preference for fish might be common, and related with either healthy or unhealthy food groups.

Global and national reports on macronutrient intake have underlined dramatic diversity across nations and the need for inform policies to improve global health. Our estimates for dietary fats are slightly “healthier” than those previously reported in the Italian population (i.e., lower cholesterol and saturated fatty acid intake) [35]; however, no previous data on specific subgroup of fats (i.e., omega-6, omega-3, etc.) or protein (plant protein, animal protein) has been reported for the Italian population. When comparing our data to global consumption of fat, we reported lower intake of dietary cholesterol (187 mg/day vs. 228 mg/day), higher of seafood omega-3 (0.53 g/day vs. 0.16 g/day) and similar of plant omega-3 (1.17 g/day vs. 1.37 g/day) [36]. Comparative data on type of protein is harder to retrieve. By roughly converting our estimates as percentage of total energy (%E), we may consider that the population investigated in this study consumed an average 5%E of animal protein (not including dairy protein) and about 9%E of plant protein: cohort studies conducted in the United States reported animal and plant protein intake of about 14% and 6%, respectively [15]; another Australian cohort reported slightly lower median intake of animal protein (about 10% of total energy) and similar of plant protein (about 6.5%) [37,38]. Thus, despite that studies to compare our reports to are scarce, we found a pattern of protein source intake healthier than in the aforementioned countries. These data on macronutrients, together with the aforementioned findings on major food groups, reflects the other findings on adherence to dietary recommendations. Various studies across the globe have reported an overall poor adherence to dietary guidelines of adult populations. Recent reports showed that diet quality of Americans, measured as agreement with dietary recommendations listed in the Healthy Eating Index (HEI), were far from optimal, regardless of socioeconomic status and race. Similarly, comparable trends have been observed in European countries. In Spain, there is a general low adherence to dietary guidelines, and these trends are particularly evident in individuals with overweight and obesity [39]. Nutrition surveys from France [40] and Germany [41] reported that consumption of fruit and vegetable does not meet dietary recommendations: similar findings were showed in other studies, where only about 30% of people living in United Kingdom [42] and 10% in Italy [43] reported eating the recommended five portions of fruits and vegetables per day. A report from Eastern European countries showed that roughly half individuals met WHO criteria for fruit and vegetable consumption, but only a minority met those for pulses and nut consumption [44]. By contrast, we found that half to two-thirds of the participants in our cohort met dietary guidelines on macronutrient and food group consumption, with the exception for total fats. However, despite that most of the individuals were under or, most likely, over the recommended intake, the results are not necessarily alarming, as we reported a higher intake of healthy rather than unhealthy fats. It has been shown that food sources of fat, such as olive oil, fish, and nuts, are associated with positive outcomes for health and a general recommendation in limiting total dietary fats may not entirely reflect a proper advice [45,46,47].

In this study, we found a correlation between certain macronutrients and food groups with BMI levels of the participants. Mostly in line with expectations, among dietary proteins, only plant protein intake was inversely correlated with BMI, while among dietary fats, monounsaturated and omega-6 fatty acids were inversely correlated, whereas trans fatty acids and cholesterol were directly correlated. However, these results did not entirely fit with correlations obtained with major food groups, as processed and unprocessed meats were indirectly and directly correlated with BMI levels, respectively. A possible reason for such unexpected findings may be the relative good quality of processed meat in southern Italy, which according the results of individual questions of the FFQ, we found it mostly referred to cured meat rather than fast foods (data not shown). Another explanation is the relatively low magnitude of the correlation for protein and meat products, which in fact might be spurious. Regarding the findings on dietary fats, we hypothesize that a major contributor to monounsaturated fatty acid intake was olive oil, highly consumed in this cohort as reported in previous studies [48]. General high levels of adherence to the Mediterranean diet has been previously shown in this cohort, as well as the association with lower likelihood of being obese and other metabolic conditions for those participants highly adherent to this dietary pattern; however, the association was not driven by olive oil or any other of the components of the score [49,50,51]. These findings corroborate the results of several other studies and suggest that the overall dietary pattern was more descriptive for a healthy nutritional alternative associated with better metabolic health [52,53,54]. Possible mediating factors have been hypothesized to be dietary polyphenols, which have been reported to exert potential beneficial effects on health [55,56]. With special regards to metabolic outcomes, dietary polyphenols have been shown to mediate, at least in part, the observed association with better metabolic health in this cohort [57,58,59]. Further studies are needed to investigate whether such compounds may explain, from a mechanistic point of view, the beneficial effects of healthy dietary pattern rich in fruit and vegetable, and other features typical of the Mediterranean diet.

The results presented in this study should be considered in light of methodological limitations. The use of FFQs is a widely-consolidated methodology, but they are also known to only provide estimates and not true intake, as they are subject to recall bias and over- and underestimation, depending on the number of food items included and social desirability bias, respectively. However, comparative reports used similar methodology and results are generally in line with literature and expected findings.

## 5. Conclusions

In conclusion, the present study provided updated information on macronutrient and major food group intake in a southern Italian adult population, taking into account specific subgroup of macronutrients rarely reported in current literature. The overall findings suggest that relatively healthy dietary habits are common in southern Italy, in up to two-thirds of the sample investigated. Further in-depth studies are needed to better understand whether findings related to foods may translate in equally adequate micronutrient intake in this cohort. However, further efforts should be made to improve diet quality of the remaining population in order to prevent non-communicable diseases.

## Figures and Tables

**Table 1:** Total, sex, and age group-specific consumption of macronutrients and fiber in the study participants of the Mediterranean healthy eating, ageing, and lifestyle (MEAL) study (*n* = 1838). * denotes *p* < 0.05.

	Total			<20 years			20 < years < 50			50 < years < 70			>70 years		
	*n*	Mean (SE)	Median (Range)	*n*	Mean (SE)	Median (Range)	*n*	Mean (SE)	Median (Range)	*n*	Mean (SE)	Median (Range)	*n*	Mean (SE)	Median (Range)
Total Energy															
Total	1838	2022.80 (15.30)	1927.63 (1000.80, 4974.01)	53	2037.36 (79.24)	1986.05 (1025.75, 3728.68)	963	2027.51 (21.91)	1930.64 (1000.80, 4865.38)	597	2038.93 (27.00)	1920.70 (1015.33, 4974.01)	225	1956.43 (36.66)	1923.88 (1010.31, 3379.32)
Men	772	2054.16 (25.38)	1939.18 (1012.84, 4974.01)	30	2101.73 (111.24)	2048.38 (1025.75, 3728.68)	384	2047.09 (37.99)	1907.56 (1012.84, 4865.38)	265	2076.45 (43.57)	1938.57 (1015.33, 4974.01)	93	2004.48 (56.27)	2025.94 (1019.88, 3334.93)
Women	1066	2000.09 (18.90)	1915.60 (1000.80, 3915.46)	23	1953.41 (111.15)	1873.20 (1133.85, 3547.77)	579	2014.52 (26.35)	1942.98 (1000.80, 3915,46)	332	2008.99 (33.84)	1902.58 (1016.84, 3911.26)	132	1922.58 (48.27)	1887.88 (1010.31, 3379.32)
Saturated fat															
Total	1838	23.58 (0.23)	22.17 (6.49, 80.45)	53	25.55 (1.29)	26.51 (11.85, 55.77)	963	23.71 (0.33)	22.11 (6.49, 80.45)	597	23.52 (0.41)	21.88 (6.91, 74.26)	225	22.69 (0.55)	22.87 (6.60, 45.05)
Men	772	23.86 (0.38)	22.09 (6.60, 80.45)	30	27.89 (1.80)	26.80 (14.82, 55.77)	384	23.45 (0.55)	20.88 (8.61, 80.45)	265	24.04 (0.64)	22.20 (9.47, 74.26)	93	23.75 (0.88)	24.47 (6.60, 44.82)
Women	1066	23.37 (0.29)	22.33 (6.49, 62.82)	23	22.50 (1.64)	21.09 (11.85, 34.77)	579	23.88 (0.40)	23.00 (6.49, 62.82)	332	23.11 (0.52)	21.46 (6.91, 61.79)	132	21.94 (0.69)	21.65 (6.97, 45.05)
Monounsaturated fat															
Total	1838	25.30 (0.21)	24.13 (7.29, 93.85)	53	27.41 (1.23)	25.73 (13.19, 63.32)	963	25.39 (0.29)	24.04 (7.29, 80.32)	597	25.28 (0.38)	23.99 (11.01, 93.85)	225	24.48 (0.49)	24.07 (7.61, 47.14)
Men	772	25.62 (0.36)	23.94 (7.61, 93.85)	30	29.12 (1.83)	26.56 (15.20, 63.32)	384	25.12 (0.51)	23.19 (11.71, 80.32)	265	25.98 (0.66)	24.26 (11.77, 93.85)	93	25.56 (0.80)	26.43 (7.61, 47.14)
Women	1066	25.07 (0.25)	24.19 (7.29, 62.38)	23	25.18 (1.45)	24.10 (13.19, 41.50)	579	25.57 (0.35)	24.74 (7.29, 62.38)	332	24.72 (0.43)	23.67 (11.01, 55.05)	132	23.71 (0.60)	23.04 (11.03, 44.77)
Total omega-6 fatty acids															
Total	1838	9.92 (0.10)	9.21 (3.08, 55.51)	53	10.33 (0.61)	8.94 (3.36, 27.60)	963	9.97 (0.14)	9.21 (3.08, 30.64)	597	10.03 (0.19)	9.22 (3.29, 55.51)	225	9.29 (0.21)	9.13 (3.64, 20.44)
Men	772	9.99 (0.16)	9.09 (3.08, 55.51)	30	10.49 (0.86)	9.43 (3.36, 27.60)	384	9.93 (0.23)	8.96 (3.08, 29.06)	265	10.19 (0.31)	9.18 (3.29, 55.51)	93	9.54 (0.30)	9.43 (4.01, 16.09)
Women	1066	9.87 (0.12)	9.27 (3.61, 32.07)	23	10.11 (0.89)	8.92 (4.89, 23.60)	579	10.00 (0.17)	9.37 (3.61, 30.64)	332	9.91 (0.23)	9.24 (3.69, 32.07)	132	9.12 (0.29)	9.03 (3.64, 20.44)
Seafood omega-3 fat															
Total	1838	0.53 (0.01)	0.38 (0.00, 5.24)	53	0.55 (0.05)	0.42 (0.00, 1.56)	963	0.52 (0.02)	0.36 (0.00, 5.24)	597	0.56 (0.02)	0.41 (0.01, 5.06)	225	0.53 (0.03)	0.43 (0.03, 3.26)
Men	772	0.53 (0.02)	0.38 (0.00, 5.24)	30	0.49 (0.07)	0.36 (0.00, 1.34)	384	0.50 (0.03)	0.34 (0.00, 5.24)	265	0.58 (0.04)	0.44 (0.05, 5.06)	93	0.56 (0.06)	0.42 (0.05, 3.26)
Women	1066	0.53 (0.02)	0.39 (0.00, 3.97)	23	0.63 (0.08)	0.56 (0.17, 1.56)	579	0.53 (0.02)	0.38 (0.00, 3.97)	332	0.54 (0.03)	0.38 (0.01, 3.05)	132	0.51 (0.04)	0.43 (0.03, 2.98)
Plant omega-3 fat															
Total	1838	1.17 (0.01)	1.06 (0.39, 5.66)	53	1.23 (0.08)	1.08 (0.51, 3.78)	963	1.17 (0.02)	1.06 (0.41, 5.51)	597	1.20 (0.02)	1.06 (0.42, 5.66)	225	1.10 (0.03)	1.05 (0.39, 2.79)
Men	772	1.17 (0.02)	1.06 (0.41, 4.48)	30	1.25 (0.11)	1.09 (0.60, 3.78)	384	1.17 (0.03)	1.02 (0.41, 3.93)	265	1.18 (0.03)	1.07 (0.46, 4.48)	93	1.14 (0.04)	1.13 (0.46, 2.32)
Women	1066	1.18 (0.02)	1.06 (0.39, 5.66)	23	1.20 (0.12)	0.90 (0.51, 2.82)	579	1.18 (0.02)	1.09 (0.42, 5.51)	332	1.21 (0.03)	1.05 (0.42, 5.66)	132	1.08 (0.04)	1.02 (0.39, 2.79)
Trans fatty acid															
Total	1838	32.31 (0.28)	30.83 (10.30, 135.12)	53	34.60 (1.69)	31.68 (16.68, 84.81)	963	32.38 (0.39)	30.82 (10.30, 100.42)	597	32.46 (0.50)	31.02 (12.26, 135.12)	225	31.09 (0.62)	30.07 (10.99, 59.82)
Men	772	32.63 (0.47)	30.63 (10.99, 135.12)	30	36.18 (2.50)	32.79 (17.03, 84.81)	384	32.05 (0.66)	29.66 (11.97, 100.42)	265	33.19 (0.88)	31.42 (12.26, 135.12)	93	32.26 (1.00)	32.83 (10.99, 59.82)
Women	1066	32.08 (0.33)	30.87 (10.30, 84.75)	23	32.55 (2.12)	30.14 (16.68, 59.93)	579	32.60 (0.47)	31.38 (10.30, 84.75)	332	31.88 (0.58)	30.35 (14.88, 68.99)	132	30.28 (0.78)	29.62 (13.84, 55.51)
Dietary cholesterol															
Total	1838	187.55 (1.93)	175.00 (17.29, 921.07)	53	198.15 (9.17)	191.00 (87.71, 371.79)	963	187.15 (2.74)	173.89 (17.29, 921.07)	597	188.24 (3.44)	172.62 (59.94, 876.81)	225	184.84 (4.92)	180.36 (42.92, 521.31)
Men	772	191.35 (3.22)	174.09 (42.92, 921.07)	30	206.26 (12.70)	206.42 (102.85, 371.79)	384	186.61 (4.64)	164.24 (56.99, 921.07)	265	195.15 (5.78)	176.70 (63.53, 876.81)	93	195.29 (7.83)	189.94 (42.92, 521.31)
Women	1066	184.80 (2.37)	176.30 (17.29, 594.94)	23	187.57 (13.09)	177.00 (87.71, 333.42)	579	187.51 (3.35)	181.67 (17.29, 594.94)	332	182.73 (4.10)	164.69 (59.94, 487.38)	132	177.65 (6.26)	169.40 (56.35, 475.25)
Total protein															
Total	1838	83.98 (0.66)	80.02 (29.27, 332.66)	53	86.24 (3.14)	83.52 (33.65, 138.33)	963	84.27 (0.96)	79.31 (29.27, 332.66)	597	83.89 (1.14)	80.18 (29.35, 303.04)	225	82.41 (1.63)	80.23 (29.35, 185.58)
Men	772	85.22 (1.11)	79.67 (33.65, 332.66)	30	88.27 (4.03)	89.30 (33.65, 137.17)	384	85.01 (1.63)	79.21 (43.56, 332.66)	265	85.59 (1.96)	79.54 (37.92, 303.04)	93	84.07 (2.56)	80.73 (39.51, 185.58)
Women	1066	83.07 (0.81)	80.23 (29.27, 215.96)	23	83.59 (5.01)	78.86 (50.69, 138.33)	579	83.78 (1.17)	80.38 (29.27, 215.96)	332	82.53 (1.32)	80.24 (29.35, 185.93)	132	81.25 (2.12)	79.47 (29.35, 156.18)
Animal protein															
Total	1838	25.65 (0.44)	22.75 (0.00, 449.25)	53	30.68 (1.83)	30.97 (6.63, 68.99)	963	26.03 (0.68)	23.08 (0.00, 449.25)	597	25.73 (0.74)	22.63 (0.00, 238.85)	225	22.67 (0.77) *	18.98 (3.05, 61.61)
Men	772	26.46 (0.67)	23.07 (0.00, 238.85)	30	31.26 (2.85)	29.70 (6.63, 68.99)	384	26.88 (0.91)	23.55 (0.00, 161.09)	265	26.46 (1.32)	23.20 (0.00, 238.85)	93	23.13 (1.09)	20.69 (6.23, 54.31)
Women	1066	25.07 (0.59)	22.38 (0.00, 449.25)	23	29.93 (2.08)	31.34 (7.14, 47.55)	579	25.46 (0.95)	22.91 (0.00, 449.25)	332	25.15 (0.81)	22.19 (5.25, 148.42)	132	22.35 (1.07)	18.71 (3.05, 61.61)
Dairy protein															
Total	1838	14.01 (0.21)	12.24 (0.00, 67.63)	53	14.84 (1.02)	12.69 (0.00, 28.48)	963	14.19 (0.30)	11.78 (0.00, 67.63)	597	13.75 (0.34)	12.85 (0.00, 52.81)	225	13.69 (0.53)	13.09 (0.00, 50.39)
Men	772	13.59 (0.32)	11.60 (0.00, 67.63)	30	13.69 (1.55)	11.91 (0.00, 28.48)	384	13.70 (0.52)	10.76 (0.00, 67.63)	265	13.17 (0.48)	13.08 (0.00, 44.02)	93	14.28 (0.78)	15.25 (0.00, 42.42)
Women	1066	14.31 (0.27)	12.71 (0.00, 54.33)	23	16.35 (1.17)	15.53 (8.52, 27.50)	579	14.52 (0.37)	12.77 (0.00, 54.33)	332	14.21 (0.47)	12.39 (0.00, 52.81)	132	13.27 (0.72)	12.04 (0.00, 50.39)
Plant protein															
Total	1838	44.71 (0.41,)	41.90 (6.90, 178.86)	53	44.13 (1.89)	43.91 (6.90, 86.56)	963	45.11 (0.61)	41.92 (13.67, 178.86)	597	44.69 (0.70)	41.88 (11.96, 140.10)	225	43.16 (0.99)	40.57 (15.11, 83.01)
Men	772	45.35 (0.67)	42.14 (6.90, 178.86)	30	44.75 (2.53)	45.04 (6.90, 70.78)	384	45.93 (1.02)	42.46 (13.67, 178.86)	265	45.19 (1.11)	41.79 (16.35, 140.10)	93	43.64 (1.55)	40.55 (17.71, 82.61)
Women	1066	44.24 (0.52)	41.80 (11.96, 117.03)	23	43.32 (2.91)	40.89 (25.91, 86.56)	579	44.57 (0.74)	41.69 (16.36, 117.03)	332	44.29 (0.89)	42.31 (11.96, 100.77)	132	42.82 (1.30)	40.62 (15.11, 83.01)
Total carbohydrates															
Total	1838	296.02 (2.56)	274.18 (100.18, 897.76)	53	289.04 (13.47)	275.29 (119.11, 590.50)	963	296.17 (3.69)	271.23 (109.87, 897.76)	597	300.52 (4.44)	278.50 (100.18, 673.97)	225	285.14 (6.31)	268.17 (114.52, 560.82)
Men	772	300.69 (4.13)	278.62 (109.87, 897.76)	30	290.46 (17.43)	275.52 (119.11, 482.17)	384	302.36 (6.33)	276.32 (109.87, 897.76)	265	303.96 (6.78)	286.63 (126.94, 673.97)	93	287.83 (9.23)	269.57 (132.74, 504.86)
Women	1066	292.64 (3.25)	270.39 (100.18, 670.72)	23	287.19 (21.57)	270.44 (137.86, 590.50)	579	292.06 (4.47)	270.50 (112.30, 670.72)	332	297.77 (5.86)	272.22 (100.18, 608.56)	132	283.24 (8.60)	265.18 (114.52, 560.82)
Fiber															
Total	1838	31.69 (0.33)	29.30 (2.81, 150.50)	53	29.77 (1.55)	27.98 (2.81, 57.65)	963	31.81 (0.50)	29.11 (6.63, 150.50)	597	32.09 (0.54)	30.43 (5.46, 100.03)	225	30.53 (0.79)	29.26 (8.31, 81.77)
Men	772	32.25 (0.54)	29.52 (2.81, 150.50)	30	30.87 (2.18)	28.23 (2.81, 57.65)	384	32.23 (0.86)	28.34 (9.48, 150.50)	265	32.65 (0.86)	30.39 (8.83, 100.03)	93	31.65 (1.14)	30.36 (11.01, 57.97)
Women	1066	31.28 (0.42)	29.23 (5.46, 85.11)	23	28.34 (2.17)	26.42 (12.77, 56.68)	579	31.54 (0.60)	29.28 (6.63, 85.11)	332	31.63 (0.68)	30.46 (5.46, 78.36)	132	29.73 (1.08)	27.75 (8.31, 81.77)

**Table 2:** Total, sex, and age group-specific consumption of animal food groups in the study participants of the MEAL study (*n* = 1838). * denotes *p* < 0.05, ** denotes *p* < 0.001.

	Total			<20 years			20 < years < 50			50 < years < 70			>70 years		
	*n*	Mean (SE)	Median (Range)	*n*	Mean (SE)	Median (Range)	*n*	Mean (SE)	Median (Range)	*n*	Mean (SE)	Median (Range)	*n*	Mean (SE)	Median (Range)
Total processed meats															
Total	1838	15.99 (0.43) **	11.50 (0.00, 168.00)	53	16.82 (1.92)	17.05 (0.00, 53.00)	963	17.57 (0.58)	11.50 (0.00, 129.50)	597	14.54 (0.82)	7.00 (0.00, 168.00)	225	12.85 (0.99)	7.00 (0.00, 157.00)
Men	772	18.00 (0.75)	11.50 (0.00, 168.00)	30	18.68 (2.67)	18.00 (0.00, 53.00)	384	19.12 (1.02)	11.50 (0.00, 129.50)	265	17.63 (1.49)	11.50 (0.00, 168.00)	93	14.24 (1.36)	7.85 (0.00, 50.00)
Women	1066	14.52 (0.49)	7.42 (0.00, 157.00)	23	14.40 (2.73)	7.00 (1.50, 53.00)	579	16.54 (0.68)	11.50 (0.00, 99.35)	332	12.08 (0.84)	7.00 (0.00, 129.50)	132	11.87 (1.39)	7.00 (0.00, 157.00)
Unprocessed meats															
Total	1838	33.78 (0.59)	28.00 (0.00, 286.00)	53	38.01 (4.06)	28.00 (0.00, 114.00)	963	33.58 (0.76)	28.00 (0.00, 136.00)	597	33.78 (1.10)	28.00 (0.00, 286.00)	225	33.67 (1.85)	28.00 (0.00, 164.00)
Men	772	34.65 (0.65)	28.00 (0.00, 286.00)	30	35.06 (5.18)	28.00 (0.00, 100.00)	384	33.58 (1.19)	28.00 (0.00, 128.00)	265	36.23 (1.80)	28.00 (0.00, 286.00)	93	34.40 (2.96)	28.00 (3.00, 164.00)
Women	1066	33.16 (0.75)	28.00 (0.00, 164.00)	23	41.86 (6.51)	28.00 (0.00, 114.00)	579	33.58 (0.99)	28.00 (0.00, 136.00)	332	31.82 (1.34)	28.00 (0.00, 136.00)	132	33.16 (2.36)	28.00 (0.00, 164.00)
Total seafood															
Total	1838	60.81 (1.28)	47.40 (0.00, 784.70)	53	60.35 (5.30)	54.70 (0.00, 145.00)	963	59.17 (1.85)	45.00 (0.00, 784.70)	597	63.71 (2.19)	50.40 (0.00, 442.00)	225	60.26 (3.45)	48.10 (0.00, 448.00)
Men	772	61.07 (2.18)	46.80 (0.00, 784.70)	30	56.56 (7.47)	46.50 (0.00, 142.00)	384	58.41 (3.28)	43.30 (0.00, 784.70)	265	65.84 (3.55)	54.10 (3.00, 442.00)	93	59.91 (6.03)	45.00 (6.00, 448.00)
Women	1066	60.62 (1.55)	48.00 (0.00, 408.00)	23	65.29 (7.40)	58.40 (12.70, 145.00)	579	59.68 (2.18)	47.40 (0.00, 408.00)	332	62.01 (2.72)	47.70 (0.00, 373.70)	132	60.50 (4.09)	50.45 (0.00, 250.00)
Eggs															
Total	1838	2.38 (0.11) *	0.77 (0.00, 24.75)	53	1.84 (0.38)	0.77 (0.00, 13.75)	963	1.92 (0.12)	0.77 (0.00, 24,75)	597	2.80 (0.22)	0.77 (0.00, 24.75)	225	3.30 (0.38) **	0.77 (0.00, 24.75)
Men	772	2.04 (0.14)	0.77 (0.00, 24.75)	30	1.72 (0.48)	0.77 (0.00, 13.75)	384	1.42 (0.11)	0.77 (0.00, 24.75)	265	2.27 (0.27)	0.77 (0.00, 24.75)	93	4.08 (0.67)	1.98 (0.00, 24.75)
Women	1066	2.62 (0.16)	0.77 (0.00, 24.75)	23	2.00 (0.61)	0.77 (0.16, 13.75)	579	2.26 (0.19)	0.77 (0.00, 24.75)	332	3.23 (0.32)	0.77 (0.00, 24.75)	132	2.75 (0.44)	0.77 (0.00, 24.75)
Cheese															
Total	1838	53.45 (0.80)	46.70 (0.00, 328.01)	53	56.29 (4.27)	50.20 (15.51, 147.47)	963	53.68 (1.13)	46.82 (0.00, 310.01)	597	53.49 (1.43)	46.08 (0.00, 328.01)	225	51.74 (2.02)	46.33 (0.00, 231.88)
Men	772	55.16 (1.30)	47.53 (0.00, 328.01)	30	64.43 (5.02)	52.67 (26.48, 147.47)	384	53.49 (1.86)	44.85 (1.50, 310.01)	265	56.61 (2.35)	48.63 (0.00, 328.01)	93	54.98 (3.13)	52.08 (0.00, 123.20)
Women	1066	52.22 (1.01)	45.82 (0.00, 296.01)	23	45.69 (6.84)	31.43 (15.51, 138.88)	579	53.80 (1.42)	47.50 (0.00, 296.01)	332	51.00 (1.74)	43.82 (0.00, 213.71)	132	49.46 (2.64)	45.72 (0.00, 231.88)
Yoghurt															
Total	1838	28.79 (1.07)	8.38 (0.00, 312.50)	53	37.23 (9.20)	8.38 (0.00, 312.50)	963	26.83 (1.36)	8.38 (0.00, 312.50)	597	29.71 (1.88)	8.38 (0.00, 312.50)	225	32.77 (3.57)	8.38 (0.00, 312.50)
Men	772	28.27 (1.66)	8.38 (0.00, 312.50)	30	50.05 (14.85)	17.50 (0.00, 312.50)	384	26.31 (2.09)	8.38 (0.00, 312.50)	265	27.70 (2.79)	8.38 (0.00, 312.50)	93	30.96 (5.28)	8.38 (0.00, 312.50)
Women	1066	29.17 (1.40)	8.38 (0.00, 312.50)	23	20.51 (7.72)	0.00 (0.00, 125.00)	579	27.18 (1.79)	8.38 (0.00, 312.50)	332	31.32 (2.55)	8.38 (0.00, 312.50)	132	34.04 (4.83)	8.38 (0.00, 312.50)
Reduced fat milk															
Total	1838	124.71 (3.68)	90.00 (0.00, 1125.00)	53	129.83 (19.42)	90.00 (0.00, 625.00)	963	127.22 (5.16)	90.00 (0.00, 1125,00)	597	118.01 (6.28)	90.00 (0.00, 1125.00)	225	130.56 (10.82)	90.00 (0.00, 625.00)
Men	772	121.70 (5.48)	90.00 (0.00, 1125.00)	30	125.20 (20.49)	90.00 (0.00, 250.00)	384	130.70 (8.31)	90.00 (0.00, 1125.00)	265	107.36 (8.60)	35.00 (0.00, 625.00)	93	124.31 (15.55)	90.00 (0.00, 625.00)
Women	1066	126.89 (4.95)	90.00 (0.00, 1125.00)	23	135.87 (36.48)	90.00 (0.00, 625.00)	579	124.91 (6.59)	90.00 (0.00, 625.00)	332	126.52 (8.94)	90.00 (0.00, 1125.00)	132	134.96 (14.88)	90.00 (0.00, 625.00)

**Table 3:** Total, sex, and age group-specific consumption of plant food groups in the study participants of the MEAL study (*n* = 1838). * denotes *p* < 0.05.

	Total			<20 years			20 < years < 50			50 < years < 70			>70 years		
	*n*	Mean (SE)	Median (Range)	*n*	Mean (SE)	Median (Range)	*n*	Mean (SE)	Median (Range)	*n*	Mean (SE)	Median (Range)	*n*	Mean (SE)	Median (Range)
Fruits															
Total	1838	395.92 (7.43)	295.13 (0.00, 2801.47)	53	335.51 (28.08)	303.58 (0.00, 951.57)	963	402.02 (11.09)	295.09 (0.00, 2801.47)	597	412.67 (12.52)	318.32 (0.00, 1822.92)	225	339.64 (16.42)	268.78 (0.00, 1545.11)
Men	772	410.55 (11.81)	305.19 (0.00, 2801.47)	30	375.82 (44.50)	326.02 (0.00, 951.57)	384	408.13 (18.20)	302.11 (0.00, 2801.47)	265	430.97 (19.18)	308.70 (0.60, 1822.92)	93	373.56 (27.59)	289.75 (18.08, 1207.35)
Women	1066	385.33 (9.54)	285.57 (0.00, 2305.08)	23	282.93 (25.86)	257.78 (72,56, 541.09)	579	397.96 (13.95)	291.35 (0.00, 2305.08)	332	398.06 (16.50)	320.91 (0.00, 1791.90)	132	315.74 (19.98)	253.81 (0.00, 1545.11)
Non-starchy vegetables															
Total	1838	219.48 (3.22)	195.86 (0.00, 1506.75)	53	250.47 (28.28)	192.78 (1.13, 1236.37)	963	214.51 (4.54)	189.54 (0.00, 1506.75)	597	222.71 (5.29)	199.63 (0.00, 1254.12)	225	224.87 (8.58)	217.68 (0.00, 1268.28)
Men	772	221.43 (5.04)	195.80 (0.00, 1506.75)	30	254.14 (31.47)	204.14 (1.13, 799.94)	384	211.29 (7.58)	182.75 (0.00, 1506.75)	265	227.06 (7.88)	203.94 (1.50, 709.37)	93	236.69 (12.51)	235.67 (36.69, 567.68)
Women	1066	218.07 (4.19)	195.86 (0.00, 1268.28)	23	245.68 (51.48)	183.20 (33.78, 1236.37)	579	216.65 (5.63)	192.75 (0.00, 1146.28)	332	219.23 (7.15)	197.86 (0.00, 1254.12)	132	216.54 (11.56)	208.32 (0.0, 1268.28)
Other starchy vegetables															
Total	1838	16.33 (0.44)	14.00 (0.00, 450.90)	53	15.88 (2.06)	14.00 (0.00, 66.00)	963	17.24 (0.72)	14.00 (0.00, 450.90)	597	15.22 (0.57)	14.00 (0.00, 130.00)	225	15.46 (0.87)	14.00 (0.00, 66.00)
Men	772	17.07 (0.80)	14.00 (0.00, 450.90)	30	17.08 (2.94)	14.45 (0.00, 66.00)	384	18.04 (1.43)	14.00 (0.00, 450.90)	265	16.03 (0.93)	14.00 (0.00, 130.00)	93	16.00 (1.34)	14.00 (0.00, 66.00)
Women	1066	15.80 (0.48)	14.00 (0.00, 130.00)	23	14.31 (2.85)	8.71 (0.00, 46.80)	579	16.72 (0.73)	14.00 (0.00, 130.00)	332	14.57 (0.72)	14.00 (0.00, 74.80)	132	15.08 (1.14)	14.00 (0.00, 66.00)
Beans and legumes															
Total	1838	35.61 (0.88)	23.70 (0.00, 655.33)	53	35.35 (4.78)	23.10 (0.00, 130.23)	963	36.69 (1.32)	24.00 (0.00, 655.33)	597	33.69 (1.33)	23.70 (0.00, 325.33)	225	36.15 (2.48)	22.33 (0.00, 184.00)
Men	772	36.33 (1.48)	23.85 (0.00, 655.33)	30	36.18 (6.39)	27.17 (0.00, 129.00)	384	37.19 (2.33)	24.10 (0.00, 655.33)	265	34.65 (2.15)	24.70 (0.00, 325.33)	93	37.58 (4.09)	22.33 (3.00, 179.00)
Women	1066	35.09 (1.08)	23.40 (0.00, 210.70)	23	34.27 (7.35)	22.33 (5.23, 130.23)	579	36.36 (1.56)	23.70 (0.00, 210.70)	332	32.91 (1.67)	23.40 (0.00, 210.70)	132	35.14 (3.10)	22.28 (0.00, 184.00)
Nuts and seeds															
Total	1838	20.30 (0.73)	11.52 (0.00, 408.40)	53	19.77 (4.48)	9.05 (0.00, 190.00)	963	19.87 (0.98)	10.35 (0.00, 408.40)	597	21.89 (1.42)	12.75 (0.00, 408.40)	225	18.10 (1.60)	10.05 (0.00, 153.40)
Men	772	20.91 (1.21)	10.40 (0.00, 408.40)	30	23.29 (7.54)	7.71 (0.00, 190.00)	384	19.42 (1.92)	7.94 (0.00, 408.40)	265	22.69 (1.70)	13.40 (0.00, 190.00)	93	21.20 (3.05)	10.35 (0.00, 153.40)
Women	1066	19.87 (0.90)	11.70 (0.00, 408.40)	23	15.17 (3.16)	11.70 (0.00, 68.80)	579	20.16 (1.02)	12.73 (0.00, 190.00)	332	21.26 (2.17)	11.52 (0.00, 408.40)	132	15.91 (1.66)	9.92 (0.00, 101.48)
Potatoes															
Total	1838	25.52 (0.58)	17.75 (0.00, 450.75)	53	31.09 (5.13)	24.20 (0.00, 253.00)	963	25.86 (0.85)	17.75 (0.00, 450.75)	597	24.98 (0.95)	17.00 (0.00, 169.20)	225	24.21 (1.30)	17.00 (0.00, 106.70)
Men	772	26.48 (0.87)	17.75 (0.00, 180.00)	30	30.72 (4.27)	24.20 (0.00, 100.00)	384	26.17 (1.22)	17.50 (0.00, 180.00)	265	26.56 (1.61)	17.00 (0.00, 169.20)	93	26.23 (2.06)	20.70 (0.00, 103.00)
Women	1066	24.82 (0.78)	17.50 (0.00, 450.75)	23	31.56 (10.58)	20.70 (0.00, 253.00)	579	25.65 (1.16)	18.68 (0.00, 450.75)	332	23.73 (1.12)	17.00 (0.00, 136.00)	132	22.78 (1.68)	16.34 (0.00, 106.70)
Whole grains															
Total	1838	27.38 (1.19)	3.00 (0.00, 330.00)	53	26.21 (5.42)	1.01 (0.00, 151.20)	963	29.34 (1.74)	3.00 (0.00, 330.00)	597	25.90 (1.99)	3.00 (0.00, 298.70)	225	23.21 (3.01)	2.10 (0.00, 270.36)
Men	772	26.56 (1.77)	3.00 (0.00, 330.00)	30	37.73 (8.69)	9.00 (0.00, 151.20)	384	30.52 (2.78)	5.40 (0.00, 330.00)	265	20.54 (2.58)	3.00 (0.00, 298.70)	93	23.75 (4.59)	3.00 (0.00, 252.85)
Women	1066	27.97 (1.59)	2.10 (0.00, 330.00)	23	11.20 3.50	0.45 (0.00, 46.80)	579	28.55 (2.23)	3.00 (0.00, 330.00)	332	30.17 (2.91)	3.00 (0.00, 298.70)	132	22.84 (4.00)	0.73 (0.00, 270.36)
Refined grains															
Total	1838	214.10 (3.04)	184.15 (3.00, 909.26)	53	197.89 (17.80)	174.05 (3.00, 576.71)	963	210.54 (4.15)	180.89 (4.50, 909.26)	597	220.89 (5.51)	189.00 (6.70, 909.26)	225	215.19 (8.41)	185.05 (11.28, 630.03)
Men	772	217.24 (4.60)	187.39 (3.00, 909.26)	30	169.00 (20.22)	173.83 (3.00, 420.10)	384	217.19 (6.47)	186.51 (12.60, 589.85)	265	226.87 (8.25)	196.70 (11.28, 909.26)	93	205.57 (11.86)	180.31 (25.20, 541.06)
Women	1066	211.83 (4.06)	182.40 (4.50, 909.26)	23	235.58 (30.14)	173.83 (3.00, 420.10)	579	206.12 (5.41)	179.66 (4.50, 909.26)	332	216.12 (7.41)	182.60 (6.70, 696.60)	132	221.98 (11.65)	187.62 (11.28, 630.03)

**Table 4:** Pearson/Spearman correlation coefficients between major food groups intake. * denotes *p* < 0.05, ** denotes *p* < 0.001.

	Total Processed Meats	Unprocessed Red Meats	Total Seafood	Eggs	Cheese	Yoghurt	Fruits	Non-Starchy Vegetables	Potatoes	Other Starchy Vegetables	Beans and Legumes	Nuts and Seeds	Refined Grains	Whole Grains
Total processed meats	1	-	-	-	-	-	-	-	-	-	-	-	-	-
Unprocessed red meats	0.217 **	1	-	-	-	-	-	-	-	-	-	-	-	-
Total seafood	0.162 **	0.072 **	1	-	-	-	-	-	-	-	-	-	-	-
Eggs	0.003	0.179 **	0.093 **	1	-	-	-	-	-	-	-	-	-	-
Cheese	0.251 **	0.200 **	0.189 **	0.094 **	1	-	-	-	-	-	-	-	-	-
Yoghurt	−0.010	−0.032	0.145 **	0.034	0.0123 **	1	-	-	-	-	-	-	-	-
Fruits	0.004	−0.004	0.121 **	−0.010	0.074 **	0.113 **	1		-	-	-	-	-	-
Non-starchy vegetables	−0.002	−0.037	0.209 **	0.016	0.167 **	0.138 **	0.297 **	1	-	-	-	-	-	-
Potatoes	0.316 **	0.085 **	0.151 **	0.084 **	0.305 **	0.063 **	0.073 **	0.075 **	1	-	-	-	-	-
Other starchy vegetables	0.075 **	−0.029	0.203 **	0.018	0.138 **	0.073 **	0.258 **	0.399 **	0.144 **	1	-	-	-	-
Beans and legumes	0.044	0.000	0.268 **	0.041	0.108 **	0.114 **	0.203 **	0.370 **	0.052 *	0.211 **	1	-	-	-
Nuts and seeds	0.098 **	0.069 **	0.048 *	0.036	0.084 **	0.005	−0.037	0.060 **	0.080 **	−0.002	0.071 **	1	-	-
Refined grains	0.052 *	0.189 **	−0.055 *	0.154 **	0.197 **	−0.152 **	0.034	−0.029	0.055 *	−0.032	−0.017	−0.021	1	-
Whole grains	0.058 *	−0.057 *	0.109 **	−0.065 **	0.079 **	0.190 **	0.151 **	0.196 **	0.008	0.070 **	0.090 **	−0.042	−0.119 **	1

**Table 5:** Percentage of study population meeting various recommendations for macronutrients (EFSA, LARN) and selected food groups (WHO).

	Total (*n* = 1839)		Men (*n* = 772)		Women (*n* = 1066)	
	Yes, % (*n*)	No, % (*n*)	Yes, % (n)	No, % (*n*)	Yes, % (n)	No, % (*n*)
EFSA						
Total carbohydrate (45–60%E)	56.3 (1035)	43.7 (804)	56.6 (437)	43.4 (335)	56.0 (597)	44.0 (469)
Total protein (>0.83 g/kg/day)	89.7 (1649)	10.3 (190)	85.5 (660)	14.5 (112)	92.8 (989)	7.2 (77)
Total fat (20–35%E)	17.1 (315)	82.9 (1524)	17.6 (136)	82.4 (636)	16.8 (179)	83.2 (887)
Fiber (>25 g/day)	62.8 (1154)	37.2 (685)	62.7 (484)	37.3 (288)	62.9 (670)	37.1 (396)
LARN						
Total carbohydrates (40–60%E)	59.4 (1092)	40.6 (747)	59.5 (459)	40.5 (313)	59.3 (632)	40.7 (434)
Total protein (>0.90 g/kg/day)	83.9 (1543)	16.1 (296)	78.6 (607)	21.4 (165)	87.8 (936)	12.2 (130)
Total fat (20–35%E)	17.1 (315)	82.9 (1524)	17.6 (136)	82.4 (636)	16.8 (179)	83.2 (887)
Cholesterol (<300 mg/day)	91.8 (1688)	8.2 (151)	91.3 (705)	8.7 (67)	92.1 (982)	7.9 (84)
Fiber (12.6–16.7 g/1000 kcal/day)	53.5 (983)	46.5 (856)	54.3 (419)	45.7 (353)	52.9 (564)	47.1 (502)
WHO						
Fruit and vegetable (>400 g/day)	74.6 (1371)	25.4 (468)	76.4 (590)	23.6 (182)	73.2 (780)	26.8 (286)
Pulses and nuts (>30 g/day)	81.7 (1503)	18.3 (336)	82.5 (637)	17.5 (135)	81.2 (866)	18.8 (200)
Total meat (<70 g/day)	77.0 (1416)	23.0 (423)	75.4 (582)	24.6 (190)	78.1 (833)	21.9 (233)

**Table 6:** Linear association between macronutrient intake and BMI levels in the study participants of the MEAL study (*n* = 1838). * denotes *p* < 0.05, ** denotes *p* < 0.001.

	Total	Men	Women
Total carbohydrates	0.000 (0.006)	−0.002 (0.008)	−0.001 (0.008)
Total protein	−0.035 (0.016) *	−0.022 (0.025)	−0.046 (0.022) *
Animal protein	0.000 (0.006)	−0.002 (0.010)	0.002 (0.008)
Dairy protein	−0.013 (0.013)	0.002 (0.020)	−0.023 (0.018)
Plant protein	−0.049 (0.022) *	0.010 (0.033)	−0.081 (0.031) **
Saturated fat	−0.023 (0.041)	−0.108 (0.064)	0.028 (0.053)
Monounsaturated fat	−0.707 (0.138) **	−0.594 (0.212) **	−0.838 (0.184) **
Total omega-6 fatty acids	−0.657 (0.173) **	−0.722 (0.251) **	−0.647 (0.242) **
Seafood omega-3 fat	0.024 (0.356)	0.272 (0.548)	−0.204 (0.471)
Plant omega-3 fat	0.654 (0.522)	2.178 (0.879) *	−0.213 (0.748)
Trans fatty acid	0.666 (0.135) **	0.520 (0.202) *	0.807 (0.184) **
Dietary cholesterol	0.021 (0.004) **	0.015 (0.007) *	0.026 (0.006) **
Fiber	−0.013 (0.016)	−0.054 (0.024) *	0.014 (0.021)

**Table 7:** Linear association between major food group intake and BMI levels in the study participants of the MEAL study (*n* = 1838). * denotes *p* < 0.05, ** denotes *p* < 0.001.

	Total	Men	Women
Total processed meats	−0.023 (0.007) **	−0.031 (0.010) **	−0.015 (0.010)
Unprocessed meats	0.017 (0.005) **	0.014 (0.007) *	0.019 (0.006) **
Total seafood	0.004 (0.002)	0.007 (0.003) *	0.002 (0.003)
Eggs	0.039 (0.024)	0.037 (0.043)	0.042 (0.031)
Cheese	0.004 (0.004)	−0.001 (0.007)	0.008 (0.006)
Yoghurt	−0.002 (0.003)	−0.010 (0.004) *	0.003 (0.003)
Fruits	−0.001 (0.000)	−0.001 (0.001)	−0.001 (0.001)
Non-starchy vegetables	0.001 (0.001)	0.002 (0.002)	0.001 (0.001)
Potatoes	−0.005 (0.005)	−0.003 (0.009)	−0.007 (0.006)
Other starchy vegetables	0.002 (0.007)	0.004 (0.009)	−0.005 (0.011)
Beans and legumes	0.001 (0.003)	−0.008 (0.005)	0.007 (0.005)
Nuts and seeds	0.002 (0.004)	0.002 (0.006)	0.000 (0.006)
Refined grains	0.002 (0.002)	0.001 (0.002)	0.002 (0.002)
Whole grains	−0.004 (0.003)	−0.004 (0.004)	−0.003 (0.003)
